# Experiences of Asian American College Students During the COVID-19 Pandemic

**DOI:** 10.7759/cureus.76009

**Published:** 2024-12-19

**Authors:** Olivia Mauchley, Claire Dinehart, Ashley Kang, Ming Wen, Akiko Kamimura

**Affiliations:** 1 Medicine, The University of Utah, Salt Lake City, USA; 2 Environmental and Sustainability Studies, The University of Utah, Salt Lake City, USA; 3 Sociology, The University of Utah, Salt Lake City, USA

**Keywords:** asian american, college students, covid-19, racism, socio ecological model

## Abstract

The purpose of this study is to examine experiences among Asian American college students during the COVID-19 pandemic. Six focus groups were held online via Zoom (Zoom Video Communications, Inc., San Jose, USA) with a total of 21 participants in October and November 2020. The focus group guide was built upon the socio-ecological model. Some participants expressed distress when having to leave their houses for pandemic safety and discrimination reasons. The impact of discriminatory events was addressed by participants’ families. Participants emphasized the importance of communities and local organizations during the COVID-19 pandemic. Participants indicated that showing support for the Asian American community through social media and public awareness campaigns could be useful in stopping the stigma associated with the COVID-19 pandemic. Understanding the experiences of Asian American college students during the COVID-19 pandemic provides insight on how to better support Asian American communities during the pandemic.

## Introduction

Outbreaks of infectious diseases are a constant threat to the health of society [1,2]. In the last 15 years alone, there have been outbreaks of influenza A (H1N1) in 2009, Ebola virus disease in 2014, and Zika virus disease in 2007 [2,3]. Most recently, COVID-19 became a global public health issue in 2020 [2]. Infectious diseases are often associated with stigma against particular populations [4]. This discrimination can then lead to a loss of employment, strained social relationships, increased risk of violence, and impacted ability to access healthcare services [5].

Asian Americans were the subject of stigma and discrimination during the COVID-19 pandemic, which started in 2020 [6-10]. The COVID-19 pandemic increased negative perceptions, macroaggressions, and hateful acts against Asian Americans due to the link to China in the early stage of the pandemic [11]. This may be related to microaggressions which are remarks, questions, or actions against a person relating to their association with a group that is discriminated against or subject to stereotypes [12]. They often occur casually, in everyday life including life during a pandemic, without harmful intent [13]. COVID-19-related microaggressions and racism have been found to have a negative effect on the health of Asian Americans [6,14]. The decline in psychological health and well-being of Asian Americans is one example of the negative impact on health caused by the stigma associated with the COVID-19 pandemic [15].

The purpose of this research is to examine instances of experiences among Asian American college students during the COVID-19 pandemic in 2020. While previous studies examined the impact of the COVID-19 pandemic on college students in general, little is known specifically about the pandemic-related experiences of Asian American college students [16,17]. The studies that have been performed on this issue found that perceived racial discrimination had a negative effect on Asian American college students including negative mental health outcomes [18]. These negative experiences of racial discrimination and their detrimental mental health impacts likely have been exacerbated during the COVID-19 pandemic in the United States. This study is important for producing qualitative evidence crucial to our better understanding of patterns and sources of racial discrimination and the ensuing public health implications.

## Materials and methods

Setting, data collection, and participants

This qualitative study was approved by the University of Utah’s Institutional Review Board (IRB) (IRB# 00136823). Qualitative primary data were collected using virtual focus groups via video conference software called Zoom (Zoom Video Communications, Inc., San Jose, USA) in October and November of 2020, during the COVID-19 pandemic. The focus group guide (see Appendix A) was developed based on the socio-ecological model (SEM) that includes individual/interpersonal, organizational, community, and public policy levels [19,20]. The individual-level factors include personal traits or states such as demographic characteristics, physical and psychological health, behavioral patterns, and social relationships between family and friends. The organizational-level factors refer to entities that influence behavior and social relations based on implicit and explicit rules. The community-level factors include larger societal relations and the interactions between organizations. The balance between these institutions can influence behavior and the perception of behavior in individuals. Public policies laid out at the local, national, and global levels can incentivize or dissuade behaviors.

Potential participants were undergraduate students at the University of Utah, which is a flagship institution in the state, self-identified Asian American college students aged 18 and older. Participants were recruited through the Asian American Student Association and email announcements. Consent was obtained from each participant. A brief online demographic survey was collected from each participant at the beginning of each focus group. Participants received a $5 e-gift card after participation in the study from the University's resource for student research.

Data analysis

All focus groups were recorded and automatically transcribed on Zoom. The transcriptions and chat posts were used as data. Data were analyzed based on thematic analysis. The code consisted of quotes from the participants that were organized by theme. Some of the themes were identified at each level of the SEM. Two research assistants separately developed codes to ensure reliability, identifying themes and categorizing participant quotes by theme. Afterward, a third research assistant merged the codes and organized the quotes by themes separately.

## Results

Participant characteristics

Table 1 describes the socio-demographic characteristics of study participants. Six focus groups were held in October and November 2020 when COVID-19 cases were rising worldwide. There were 21 participants in total (18 females and 3 males) from convenience sampling. The self-identified ethnic groups (multiple choice if a participant reported more than one ethnic group) included Chinese (n=8), Vietnamese (n=6), Filipino (n=3), Korean (n=2), Asian Indian (n=2), Taiwanese (n=1), and Laotian (n=1). One participant was married. One participant was an international student. Out of 20 participants who were born in the U.S., only one participant did not have a foreign-born parent. More than half of the participants were 21 or 22 years old.

**Table 1:** Characteristics of participants

Characteristics	N	%
Gender		
Female	18	85.7
Male	3	14.3
Marital status		
Married	1	4.8
Single or never married	20	95.2
Ethnicity (multiple answers)		
Chinese	8	38.1
Vietnamese	6	28.6
Filipino	3	14.3
Korean	2	9.5
Asian Indian	2	9.5
Taiwanese	1	4.8
Laotian	1	4.8
International student	1	4.8
Having foreign-born parent (among 20 US born participants)	19	90.5

Individual/interpersonal level

Some participants experienced or witnessed hate acts toward Asians. For example, one Asian Indian participant stated, "With me being Indian, being Southwest Asian, and I'm not many times viewed as Asian…I'm not the one that's getting the hate directed towards me, necessarily, but I've definitely noticed that…if we have neighbors that are talking in general, a lot of times…there's just so much…hatred towards the [Asian] people themselves."

Hate toward Asians targeted Chinese people in particular. For example, one Chinese participant stated, "And I think that people have a hard time differentiating between like the Chinese people versus [other Asians] they're always saying the Chinese the Chinese the Chinese…Since this pandemic, being in my neighborhoods it's a lot of hatred towards the Chinese community. It's not so much the other Asian countries, really, it's just like, I mean obviously did come out of China, but yes, just like a solicited unnecessary hatred."

As a result, some participants did not feel comfortable going to stores or felt distressed. This impact of the pandemic was felt not only by the participants, but also by their families as well (e.g., “Um, see my mom works from home so she hasn't really felt that. She definitely got really scared to go out after the pandemic after hearing all like, you know, attacks on Asian Americans and stuff.”). Many participants’ families, especially the older family members, limited outdoor activities due to fear of the virus and caution against anti-Asian hate crimes. One participant said the following: "My grandma, she used to go outside and walk alone a lot, and now all because of all the different stories she just walks around in the backyard and she's too afraid to go anywhere else. Just in case, since she can't defend herself and if anything happens, she doesn't have a phone…"

Another participant expressed similar concerns: "It's made my family more fearful to go out alone, especially my mom and my grandma. Um, if there's no one to go to the grocery store with them or somewhere with them, then they refuse to go alone, just because they're afraid of someone hurting them."

At the same time, participants did not feel an impact from the pandemic and discrimination against Asians (e.g., “I mean it hasn't been that bad, there's definitely been like more racist comments like, it's not, I guess, inspiring and just more like micro aggressions you know like stuff like.”). Although participants did not personally witness acts of hate, they admitted that hearing about them had psychological and emotional impact. One participant described the impact of the COVID-19 pandemic on psychological well-being as “And my mental health is definitely like declining, I felt like. But I never wanted to like, recognize it, because I do come from a culture that does…”

Organizational level

Participants stated that universities can speak up for their Asian and Asian American students to provide support during COVID-19, (e.g., “I think, and you know and like speaking on behalf of or,…speaking up for the international students, Asian international students, are definitely something that the university could do.”). Furthermore, participants said that universities can also hold information sessions or incorporate learning about current events in the classroom (e.g., “doing more to help other people understand that there's a lot more going on around the world with discrimination and Asians than what people might know.”). Participants in the focus groups expressed a desire for a public show of allyship in addition to reaching out to Asian American students. Public support can be shown to the Asian American community through social media: "I really like the idea of the [the name of the university] posting something in support, because I feel like social media is really good outlet for that. So, not even necessarily policy, but just showing their support outwardly because I feel like that you can say a lot of things, but that cements it because it's more permanent."

Community level

Participants felt that the greater community placed blame on Asians for the COVID-19 pandemic (e.g., “I think most people blame the Asian nations…public pressure…[comes] from everywhere: from media, or the internet, or just people.”). Participants emphasized the importance of unity in the Asian American community during the COVID-19 pandemic (e.g., “I think the Asian community should be united, because if you do not trust your peers or the people in your community who belong to your community, it will lead to a trust crisis, and you…cannot deal with the problems in a good way. So, we need to stand together to deal with the issue.”). Another participant shared that the community can, “Try our best to protect our families and friends. From the simple sense…you can wear masks and not hold a party in…your house.”

Public policy level

Participants pointed out negligence in public policy to address anti-Asian hate crimes and attributed the idleness to the political climate in 2020 (e.g., “One of the biggest issues is discrimination and I think that is becoming something - that is something that has become and becoming bigger and bigger. Just because of the political environment we're in.”). In regards to national politics, one student said, "I think people have conformed to the idea that the President has put out, that it is the China virus, since he clearly isn't taking leadership or ownership of it. So, um, so I think, again, people, the government [don’t change] if the President doesn't change."

Another student also called out the federal government and President Trump, "President Trump has always said the ‘China virus.’ We are hurt [by], this speaking, we feel angry or sometimes we may feel ridiculous…You know, the, the situation in China is getting better than before. But hey, [he still?] talks about that and it really makes us feel angry."

Other students expressed frustration with local leaders and their inaction on discrimination at a state level. "I think government [can] do a lot more and taking ownership and leadership of what's going on and speak out on any news, or go against some of the wording of the virus, but I just think it's hard, especially in [name of state], being a red state…I just think there should be more activism and more publications…explaining what's going on. So, people actually understand that it's hurtful, that the Asian community is being harmed."

Reports of violence and discriminatory acts against Asian Americans in addition to passivity from public policymakers on the subject make students feel terrified (e.g., “You know it's terrifying to see all the news. About it and thinking that that could have been me or my family members.”). Another participant stated “[What] I'm seeing that on the news is very terrifying. And it really could have been me. My family, my friends, and any one of my close circles…seeing it on the news almost makes me feel like someone in my community is going to see that [soon].” They also indicated feeling hurt and sad (e.g., “When I saw this on news on the media. I feel kind of a sad because…I once read on news about the Asian woman who [lives] in New York, and she was attacked by the local people, and they said that it's about the Asian [who] brought the COVID-19 to them.”).

One participant shared their experience speaking out against harmful language aimed at the Asian American community in their own lives in the U.S., "As for like the wording of things like ‘Kung flu’ and the ‘China virus.’ It's kind of hurtful and whenever I do speak out to some of my peers…they end up saying that ‘it's not a big deal because there's a Spanish flu’ things like that. It’s just hard to know that there's a lot of people that are ignorant of what's going on. But it's just. it's just scary to know that there's people who just don't realize that there's so much going on with the Asian community that it's hurtful to even have those names."

The discrimination is not limited to the U.S. as another participant pointed out, "Europe usually is on this pillar of greatness and modernity and being progressive and having those issues as well, um, it’s just very saddening because these countries and these civilizations the societies that we thought were doing better…[and] more accepting aren't. You [see] people that aren't and that are perpetuating these ideas of discrimination…Makes me remember that we still need to promote change."

The negative media attention has inspired students to suggest actions to reduce the stigma surrounding COVID-19 and Asians. They offered solutions such as increasing awareness through social media posts (e.g., “I feel like awareness is the biggest part because it's hard to get everyone to understand what's going on. But the more we post the more we share information things will get a lot easier.”) and ending the use of derogatory terms (e.g., “Stop using diction, such as the China flu.”). News coverage can also focus on Asian American stories (e.g., “…including them in media coverage. I feel like would help maybe destigmatize things, or even just like give more information about what Asian Americans actually are doing…instead of just maintaining these foreign ideas of Communist China.”). Participants also believe statements from political and community leaders can contribute to ending the stigma (e.g., “I think government [can] do a lot more in taking ownership and leadership of what's going on and speak out.”).

## Discussion

This study focuses on the experiences of Asian American college students in the U.S. during the COVID-19 pandemic in 2020. There are three main findings. First, the biggest issue facing Asian American college students from this study participants is discrimination based on institutionalized racism and a negative stigma centered on the COVID-19 pandemic. Second, there were not sufficient resources to support Asian American students at the organizational and community levels during the COVID-19 pandemic, from the perspectives of the participants of this study. Third, the political climate during the COVID-19 pandemic in 2020 could be one of the main sources of discrimination against Asian American students.

Asian American college students experienced discrimination based on racism and a negative stigma centered around the COVID-19 pandemic. The results are consistent with previous studies. For example, Asian American young adults experienced COVID-19-related discrimination [14]. Discrimination against the Asian American community is often rooted in the model minority myth. The model minority myth is based on the racial stereotype that Asian Americans are polite, achieve higher levels of success, and work harder than other minorities to achieve that success [21]. This myth causes inaccurate assumptions about Asian Americans [22]. The high achievement stereotype also disregards the fact that Asian Americans are underrepresented in political and corporate leadership positions [22]. As COVID-19-related discrimination against Asian Americans was related to political rhetoric [23], the underrepresentation of Asian Americans in politics could affect discrimination against Asian American college students during the pandemic based on the perspective of this study's population.

In addition, resources to support Asian American students are lacking at the organizational and community levels during the COVID-19 pandemic. When U.S. schools reopened during the pandemic, the focus was on infection-related concerns, and the impact of discrimination against Asian and Asian American students was not addressed [24]. The increased racism during the pandemic negatively affected the psychological well-being of Asian American students [25]. In addition to discussing infection prevention during the COVID-19 pandemic, social impacts on Asian Americans should also be included in the discussion of disease management. Universities and schools should do more to educate their students about current news and updates within the Asian American community. Racial/ethnic majority group members treat the ethnic minority with more positive regard when they are presented with lower levels of discriminatory ideas against the minority [26]. Raising awareness about racism and issues such as White privilege in the classroom increased support for affirmative action and reduced prejudice and fear of other races [27]. By raising awareness among university students of the social impacts COVID-19 has on Asian Americans, Asian American college students may have a more positive experience during the COVID-19 pandemic.

Furthermore, policy-level changes to reduce racism or discrimination against Asian Americans should help enhance the well-being of Asian American college students. The Asian American Pacific Islander Organization (AAPI) states that 1900 reports of harassment, discrimination, and physical assault were filed to their “Stop AAPI Hate” over an eight-week period since March 19, 2020 [28]. In fact, the World Health Organization has strict guidelines regarding the naming of diseases because calling something the “China Virus” personifies the virus and vilifies those who identify as such. Since 2020, the U.S. government has taken action to pass a bill that targets anti-Asian hate crimes [29]. The legislation passed in the U.S. Senate with an overwhelming bipartisan vote, where it continued on to the House floor in May 2021 and was signed by Democrats on May 20, 2021 [30].

While this study contributes to research on Asian Americans and the COVID-19 pandemic, there are limitations. First, all of the participants were college students. The specific population helped to describe the perspectives of the educated young adults, their experiences may be very different from other Asian American populations. Second, all the interviews were conducted virtually. There is a possibility that potential participants who do not prefer a virtual environment to express themselves did not participate. The number of participants was relatively small. Finally, because some of the participants turned off the camera and/or were quiet on Zoom, it was difficult to assess whether all the participants were given an opportunity to share their perspectives.

## Conclusions

The sample population of this study, Asian American college students, reported distress, hurt, and sadness when speaking about anti-Asian rhetoric and hate crimes during the COVID-19 pandemic. Ending the stigma associated with the COVID-19 virus and Asian Americans can start with education campaigns, unity in the community, and public support for Asian Americans on social media. Universities also have a responsibility to educate their students on diversity with a greater focus on Asian Americans. The inaction of government institutions and a lack of community support leads Asian Americans to fear for themselves and their families during the pandemic. Understanding the experiences of Asian American college students during the COVID-19 pandemic provides valuable insight into how the greater community can better support Asian Americans and creates a more nuanced awareness of how to improve the experiences of minority groups. Future research should be done using a quantitative or a mixed method to further investigate this issue.

## Appendices

Appendix A

Focus Group Guide

My name is ______ and I am a member of the research team at the University of XXX. We are interested in learning about the experience of Asian Americans during the COVID-19 pandemic. My mentor is Dr. XXX from the department of Sociology. We expect this study to contribute to a greater understanding of discriminatory acts against Asian Americans. This study is important to ensure the well-being of Asian Americans and has potential implications for other minority groups during the COVID-19 pandemic.

Our meeting today will be video and audio recorded and transcribed for the team. You may choose to turn your video camera off for the duration of the meeting if that makes you more comfortable. Please use your first name or preferred name only during the interview. Before the interview, please read the consent cover letter and fill out and submit a brief demographic survey. I am posing the link to the survey in the chat. You will be compensated for your time with an Amazon E-gift card. In order to receive a gift card, please stay in the focus group until the end of the session. 

Opening question:

-What do you think about the pandemic situation?

Individual and Interpersonal

-Can you briefly introduce yourself- your first name and your ethnic background.

-Do you feel a strong connection to your self-identified culture and ethnicity? Why or why not?

-Could you describe your experiences as an Asian American since the pandemic started here in mid-March?

-How about your family?

-Have you ever feared for your own safety or the safety of your loved ones during this time because of acts of discrimination against Asian Americans in the country?

-How has COVID-19 affected your psychological well-being especially as an Asian American?

-If you have been negatively affected by the pandemic, how have you dealt with the struggles of living through a global pandemic?

Organizational

-What can schools/communities do to support Asian Americans?

-What resources should universities add to better help Asian students in the current pandemic situation?

Community

-What are the biggest issues that you see facing the Asian American community?

-How should the Asian community deal with the issues related to COVID-19 and discrimination?

Public Policy

-Since the start of COVID-19, reports of violence and discriminatory acts against Asians around the world have increased and been covered on the news. How does this information make you feel? How does this affect the public perception of Asian Americans? For example, the statements made by the POTUS referring to COVID-19 as the “China-virus” or “Kung-Flu”

-How can we reduce the stigma surrounding COVID-19 and Asians?

-What policy changes could be made at the local, state, or federal level to help address discrimination against Asian Americans?

Aggregate ethnic groups, specify who you are talking about, educate youth about different cultures, give funding to non-profits and the communities who need it, ethnic studies beginning in elementary

Moving Forward + Closing Reflection

-Do you have any final thoughts or feel anything was left out of the focus group?

Thank you for your participation
